# Outcomes of thoracic endovascular aortic repair with chimney technique for aortic arch diseases

**DOI:** 10.3389/fcvm.2022.868457

**Published:** 2022-08-04

**Authors:** Jiehua Li, Yunfei Xue, Shangqian Li, Likun Sun, Lunchang Wang, Tun Wang, Kun Fang, Mingyao Luo, Xin Li, Hao He, Ming Li, Quanming Li, Alan Dardik, Chang Shu

**Affiliations:** ^1^Department of Vascular Surgery, The Second Xiangya Hospital of Central South University, Changsha, China; ^2^Vascular Diseases Institute of Central South University, Changsha, China; ^3^Fuwai Hospital, National Center for Cardiovascular Disease, Chinese Academy of Medical Sciences and Peking Union Medical College, Beijing, China; ^4^Department of Vascular Surgery, School of Medicine, Yale University, New Haven, CT, United States

**Keywords:** chimney technique, thoracic endovascular aortic repair, aortic arch diseases, type IA endoleak, aortic dissection

## Abstract

**Objective:**

This study aimed to summarize the long-term experience of using the chimney technique in thoracic endovascular aortic repair (TEVAR) for aortic arch diseases.

**Methods:**

From November 2007 to June 2021, a total of 345 consecutive patients (mean age 56 ± 11.3 years, range 28–83, 302 men) with aortic arch pathologies underwent TEVAR combined with chimney technique (cTEVAR). Their medical data and follow-up results were retrospectively reviewed and analyzed.

**Results:**

Among the 345 patients, 278 (80.6%) received single chimneys, 53 (15.4%) received double chimneys, 7 (2%) received triple chimneys, and 7 (2%) underwent cTEVAR accompanied by other techniques (two with extra-anatomical bypass, two with *in situ* fenestration, and three with physician modified fenestration). A total of 412 chimney stents were used, including 27 in the innominate artery (IA), 113 in the left common carotid artery, 270 in the left subclavian artery, and two in the aberrant right subclavian artery. Early type IA endoleaks were found in 38 (11%) patients, including 12 with the double or triple chimney technique. Early type II endoleak was found in nine (2.6%) patients. Early re-intervention occurred in two patients with double chimney technique, one for chimney stent migration and the other for compression of chimney stent. The 30-day mortality was 1.2% (4 in 345). During a mean follow-up of 42 ± 22 months (range 1–108 months), major stroke occurred in nine (2.6%) patients, chimney occlusion or stenosis occurred in six (1.7%), and retrograde type A aortic dissection occurred in four (1.2%). Fourteen (4.1%) patients received the secondary intervention. The all-cause mortality was 6.7% (23 in 345). Additionally, the total adverse event rate after cTEVAR was 13.9% (48 in 345).

**Conclusion:**

TEVAR with chimney technique provides a minimally invasive alternative with good chimney graft patency and low postoperative mortality during follow-up. However, the double and triple chimney techniques should be used cautiously as they seem to have a higher risk for type IA endoleak and adverse events after the operation.

## Introduction

Thoracic endovascular” aortic repair (TEVAR), which was first used to treat thoracic aortic aneurysms in 1994, has gradually become the first-line treatment for descending aortic diseases (1). Furthermore, as experience accumulated, devices improved, and technology advanced, the indications for TEVAR have expanded. Endovascular management of lesions involving the aortic arch, which represents 30% of thoracic aortic diseases, remains challenging because of the angulated morphology and involvement of the vital supra-aortic branches. Different kinds of therapeutic strategies have been proposed and practiced to preserve supra-aortic branches in TEVAR, such as the chimney technique, fenestrated/branched stent-grafts, and hybrid procedures (TEVAR together with extra-anatomical bypass), et al. (2, 3).

Although hybrid procedures are much less invasive than open surgery, they could still lead to significant morbidities and mortalities (4, 5). Fenestrated/branched techniques have shown favorable outcomes in several studies, but these approaches are limited by the morphological diversity of aortic arch and the availability of patient-specific or tailor-made devices (6, 7). Moreover, such procedures are often not suitable in emergency situations. The chimney technique is an important option to preserve supra-aortic branches, which was first reported by Criado as a means to rescue an inadvertently covered left subclavian artery (LSA) (8). The chimney technique offers the advantage of using standard off-the-shelf devices and the manipulation is relatively simple and its use in TEVAR has expanded in recent years (9). However, the long-term efficacy of the chimney technique remains largely unknown, and the risk of type IA endoleak is the major concern that hinders its broader use in TEVAR.

The aim of our study was to report our 14-year experience with the chimney technique in the endovascular treatment of aortic arch diseases and evaluate the short-term and long-term outcomes.

## Materials and methods

### Study design and patient sample

This retrospective study was performed with the approval of the institutional ethics boards of the Second Xiangya Hospital and Fuwai Hospital (NOS. 2020S066 and D171100002920058, respectively). Informed consent was obtained from the patients and their relatives. From November 2007 to June 2021, a total of 345 patients (mean age 56 ± 11 years, range 28–83 years; 302 men) with various aortic arch pathologies underwent TEVAR with the chimney technique (cTEVAR) to reconstruct the supra-aortic branches in these two medical centers. The indications for the chimney technique were aortic arch diseases (including dissection, aneurysm, pseudoaneurysm, penetrating atherosclerotic ulcer, intramural hematoma, type IA endoleak post-TEVAR, etc.) in the high-risk patients that required TEVAR with proximal landing in zone 0, 1, or 2, or as a bailout in TEVAR. All patients received CT angiography (CTA) and were evaluated by an interdisciplinary board composed of cardiologists, endovascular surgeons, cardiovascular surgeons, radiologists, and anesthetists. The medical records of these 345 patients were retrospectively reviewed and analyzed. Aortic pathologies included acute aortic dissection in 234 (67.8%) patients, chronic aortic dissection in 21 (6.1%) patients, thoracic aortic aneurysm (TAA) in 33 (9.6%), penetrating atherosclerotic ulcer in 20 (5.6%) patients, aortic pseudoaneurysm in 3 (0.9%) patients, intramural hematoma in 22 (6.4%) patients, and type IA endoleak after TEVAR in 12 (3.5%) patients. Baseline characteristics of the patients are presented in Table 1.

**TABLE 1:** Baseline characteristics of the patients.

Variables	Number or mean	Range or %
Age, y	56.0 ± 11.3	28–83
Male	302	87.5%
Primary diagnosis		
Acute aortic dissection	234	67.8%
Chronic aortic dissection	21	6.1%
Aortic aneurysm	33	9.6%
Penetrating aortic ulcer	20	5.6%
Aortic pseudoaneurysm	3	0.9%
Intramural Hematoma	22	6.4%
Type IA endoleak post TEVAR	12	3.5%
Comorbidities		
Hypertension	300	87%
Coronary heart disease	32	9.3%
Diabetes	22	6.4%
Renal dysfunction	16	4.6%
COPD	32	9.3%
Previous stroke	26	7.5%
Marfan Syndrome	3	0.9%
Smoking	224	64.9%

TEVAR, thoracic endovascular aortic repair; COPD, chronic obstructive pulmonary disease.

### Operative procedures and device selection

As we previously reported (10–12), all procedures were performed in a hybrid operating room with fluoroscopic guidance. All patients received general anesthesia with tracheal intubation. Antihypertensive and antitachycardia therapies were initiated before the operation.

Routinely, a common femoral artery was exposed surgically, and then a 5-F calibrated angiographic pigtail catheter was inserted for digital subtraction angiography (DSA) to evaluate the aortic pathology and cerebral circulation. The left and/or right brachial artery, and/or left common left carotid artery were exposed according to the planned configuration of aortic arch reconstruction. The aortic stent-graft and the chimney stent-graft (s) were selected with 10–20% and 0–5% oversizing, respectively.

Taking the cTEVAR for LSA as an example, an 8- to 10-F sheath (according to the size of chimney graft) was placed in the exposed left brachial artery, and then a Lunderquist extra-stiff guidewire (William Cook Europe, Bjaeverskov, Denmark) was advanced into ascending aorta. Another Lunderquist extra-stiff guidewire was advanced into ascending aorta from the access at the common femoral artery. The chimney stent-graft was inserted from the brachial access into the aortic arch with the distal end in LSA. The aortic stent-graft was inserted from the femoral access and deployed in the targeted position of the aortic arch, and then the chimney stent-graft was deployed parallel to the aortic stent-graft. The chimney grafts were routinely molded with a balloon. DSA was performed to evaluate the positions of the stent-graft and the chimney stent, as well as to detect if there was any immediate endoleak. Aspirin (100 mg/day) was prescribed to patients after cTEVAR.

Specifically for the application of cTEVAR in bailout situations, the left brachial artery or left common carotid artery (LCCA) was exposed immediately when the LSA or LCCA was inadvertently covered by the aortic stent. Then an 8- to 10-F sheath was placed, and a super smooth guide wire and catheter were inserted into the ascending aorta parallel to the aortic stent. Moreover, a Lunderquist extra-stiff guidewire was advanced to ascending aorta. The chimney stent was inserted and deployed in the targeted place and was routinely molded with a balloon.

All the chimney stent-grafts in our study were covered stents, including Fluency (C.R. Bard, Inc, Murray Hill, NJ, United States) and GORE VIABAHN (W.L. Gore & Associates, Flagstaff, AZ, United States). The aortic stent-grafts included Hercules (MicroPort Medical Co, Ltd, Shanghai, China), Zenith (Cook, Inc, Bloomington, IN, United States), Ankura (Lifetech Scientific Co, Ltd, Shenzhen, China), and Valiant (Medtronic, Inc, Minneapolis, MN, United States).

### Follow-up

All of the patients were scheduled with follow-up physical examination and CTA at 2 weeks, 3, 6, and 12 months after TEVAR, and annually thereafter to evaluate the stent-graft position, endoleaks, and changes of aortic pathologies. Survival and clinical manifestations were assessed by outpatient clinic visits or telephone interviews. For patients not observed at an outpatient clinic, the information was obtained by a telephone call to the family or the patients themselves.

### End points and outcome assessment

The primary endpoints were all-cause mortality, major complications (including stroke, cerebral hemorrhage, spinal cord ischemia, limb ischemia, retrograde type A aortic dissection et, etc.), type I or type II endoleak, and re-intervention or conversion to open repair. The secondary endpoint was stenosis or occlusion of the chimney grafts. Primary technical success was defined as successful deployment of the device to the targeted location and complete exclusion of the treated pathology in the absence of branch obstruction and significant type I or type II endoleak. The adverse events included all the end-point events but not the type I or type II endoleaks that were asymptomatic and untreated.

### Statistical analysis

Descriptive statistics were used to describe patient data and outcomes in this cohort. Continuous data are expressed as mean ± *SD* or median (range), and categorical data are given as the counts (percentage). The Kaplan–Meier analysis was used to establish the rate of freedom from all-cause death and freedom from all adverse events). *P* < 0.05 was considered statistically significant. Statistical analysis was conducted using GraphPad 7 (GraphPad Software, Inc., San Diego, CA, United States).

## Results

### Operative details

The mean operation time, fluoroscopy time, and contrast volume were 96 ± 32 min, 28 ± 18 min, and 102 ± 45 ml, respectively. TEVAR was performed in emergency settings in nine patients with acute aortic dissection that was complicated by intestinal or lower extremity ischemia. Chimney stents were deployed in six patients as bailout to reconstruct LCCA (3) or LSA (3) due to inadvertent coverage in TEVAR.

**FIGURE 1:**
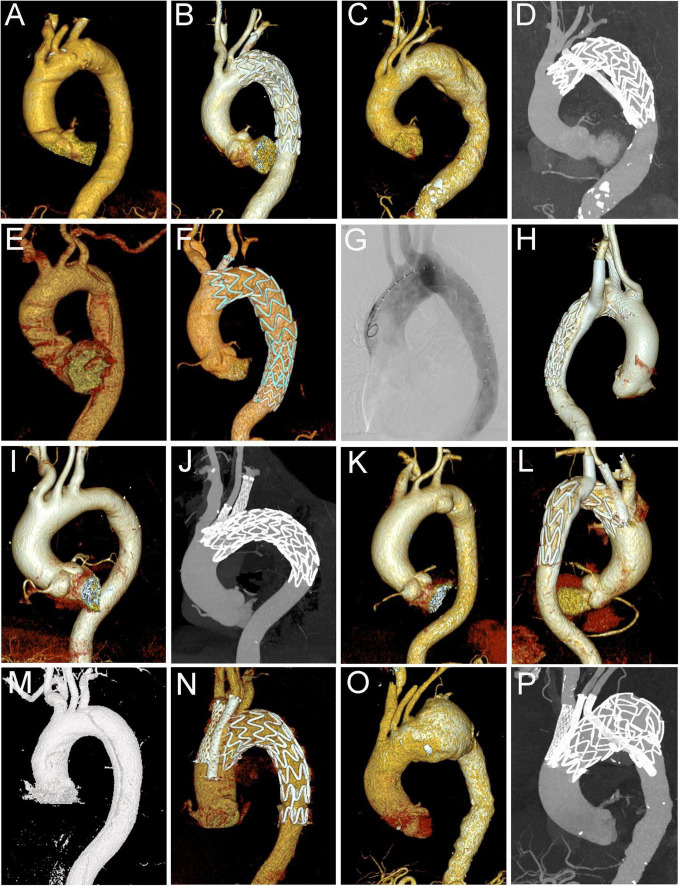
Different types of cTEVAR in the study. **(A,B)** Chimney stent in LSA; **(C,D)** snorkel stent in LSA; **(E,F)** chimney stent in LCCA; **(G,H)** chimney stent in LSA and snorkel stent in aRSA; **(I,J)** chimney stents in LCCA and LSA; **(K,L)** chimney stent in LCCA and snorkel stent in LSA; **(M,N)** chimney stents in IA and LCCA; **(O,P)** chimney stents in IA and LCCA, and snorkel stent in LSA. cTEVAR, TEVAR with chimney; IA, innominate artery; LCCA, left common carotid artery; LSA, left subclavian artery; aRSA, aberrant right subclavian artery.

As shown in Figure 1, there were different types of cTEVAR performed in our study. 278 (80.6%) patients received single chimneys, including 50 in LCCA and 228 in LSA. A total of 53 patients (15.4%) underwent the double-chimney procedure, among whom 20 had chimney stents deployed in an IA and LCCA, 31 in LCCA and LSA, and 2 in LSA and aberrant right subclavian artery (aRSA). There were seven (2%) patients who received triple chimneys in the IA, LSA, and LCCA. Besides these conventional chimney procedures, seven (2%) patients received cTEVAR accompanied with other techniques, among whom two received LCCA chimney and LSA-RSA bypass, while five received cTEVAR accompanied with fenestration (two with *in situ* fenestration and three with physician modified fenestration). Therefore, a total of 412 chimney stents were used, including 27 in IA, 113 in LCCA, 270 in LSA, and 2 in aRSA. The flow in 383 (93.%) chimney stents was in the same direction as the aortic flow, while the flow of 29 (7%) chimney stents was in the reverse direction of the aortic flow, which was also known as the “periscope” technique. The aortic stent-grafts were landed proximally in zone 0 (*n* = 27), zone 1 (*n* = 86) or zone 2 (*n* = 232). A total of 28 (8.1%) patients received more than one aortic stent-graft. Finally, six (1.7%) patients received endovascular repair of thoracic and abdominal aortic lesions simultaneously. The operative details were summarized in Table 2.

**TABLE 2:** Perioperative results.

Variables	Number or mean	Range or %
Operation time, minutes	96 ± 32	60–210
Fluoroscopy time, minutes	28 ± 18	20–72
Contrast volume	102 ± 45	80–220
Types of cTEVAR		
Single chimney	278	80.6%
Double chimney	53	15.4%
Triple chimney	7	2%
cTEVAR with other techniques	7	2%
Aortic branches with Chimneys		
IA	27	7.8%
LCCA	113	32.8%
LSA	270	78.3%
aRSA	2	0.6%
Landing zone		
0	27	7.8%
1	86	24.9%
2	232	67.2%
Early endoleaks		
IA	38	11%
IB	6	1.7%
II	9	2.6%
Early re-intervention	2	0.6%
Death within 30 days	4	1.2%

cTEVAR, TEVAR with chimney; IA, innominate artery; LCCA, left common carotid artery; LSA, left subclavian artery; aRSA, aberrant right subclavian artery.

### Thirty-day outcomes

All the aortic stent-grafts and chimney stents were implanted in the targeted locations. Angiography after chimney stent placement showed that all supra-aortic branches were patent and revealed type IA endoleaks in 38 (11%) patients, type IB endoleaks in six patients, and type II endoleaks from LSA in nine patients. Thus, the primary technical success rate was 84.6% (292 in 345). Repeated balloon molding during TEVAR nearly obliterated five type IA endoleaks, which had disappeared at the time of discharge. Three patients with type II endoleaks were treated with a duct occluder device, while other patients with endoleaks were treated conservatively with close surveillance. The rate of early-type IA endoleak was 8.6% (24 in 278) in patients with single chimney, and 20% (12 in 60) in patients with double or triple chimneys (8.6 vs. 20% *P* < 0.05). Two early-type IA endoleaks were observed in the seven patients receiving cTEVAR accompanied with other techniques.

Early re-intervention occurred in two patients, both of whom underwent the double chimney technique to reconstruct IA and LCCA. One patient presented decreased oxygen saturation and undetectable carotid pulses during recovery from general anesthesia. DSA showed distal migration of both chimney stents in IA and LCCA. Two more chimney stents were implanted emergently, and the blood flow in IA and LCCA was restored. The patient had cerebral infarction with right-sided neurological dysfunction after the operation, but he gradually recovered 3 months later. The other patient had worsening somnolence 4 days after cTEVAR due to a completely compressed chimney stent-graft in the IA. Under local anesthesia, another self-expanded bare stent was deployed within the previously placed chimney stent. The patient completely recovered within 2 days.

The 30-day mortality rate was 1.2% (4/345). The reasons for deaths were multiple organ failure in one case, cardiac arrest in one case, intestinal ischemia in one case, and gastrointestinal perforation in one case. Three major strokes were recorded in the short term (including the one who received early re-intervention). Two of these patients gradually recovered at discharge, while the other recovered 3 months later. Spinal cord ischemia occurred in one case possibly due to the long coverage of descending aorta. The patient was treated with cerebrospinal fluid drainage and his strength of lower limb muscles was partly recovered at discharge. Therefore, major adverse events occurred in 2.6% (9/345) of the patients in the short term.

### Follow-up results

Approximately 90% (310/345) of the patients were successfully followed up by outpatient clinic visits or telephone interviews to assess survival and clinical findings. The mean duration of clinical follow-up was 42 ± 22 months (range 1–108 months). The follow-up results were shown in Table 3.

**TABLE 3:** Follow-up outcomes.

Variables	Number or mean	Range or %
Patients in follow-up	310	90%
Follow-up time, months	42 ± 22	1–108
Major adverse events		
Chimney occlusion or stenosis	6	1.7%
Stent-graft migration	2	0.6%
Distal intimal tear	3	0.9%
Major stroke	9	2.6%
Spinal cord ischemia	1	0.3%
RAAD	4	1.2%
Aortic rupture	4	1.2%
Re-intervention	14	4.1%
Surgery for RAAD	3	0.9%
TEVAR for distal tear	3	0.9%
For type IA endoleak	3	0.9%
For type II endoleak	2	0.6%
For compressed chimney stents	2	0.6%
For chimney stent migration	1	0.3%
Aorta-related mortality	4	1.2%
All-cause mortality	23	6.7%

RAAD, retrograde type A aortic dissection.

**FIGURE 2:**
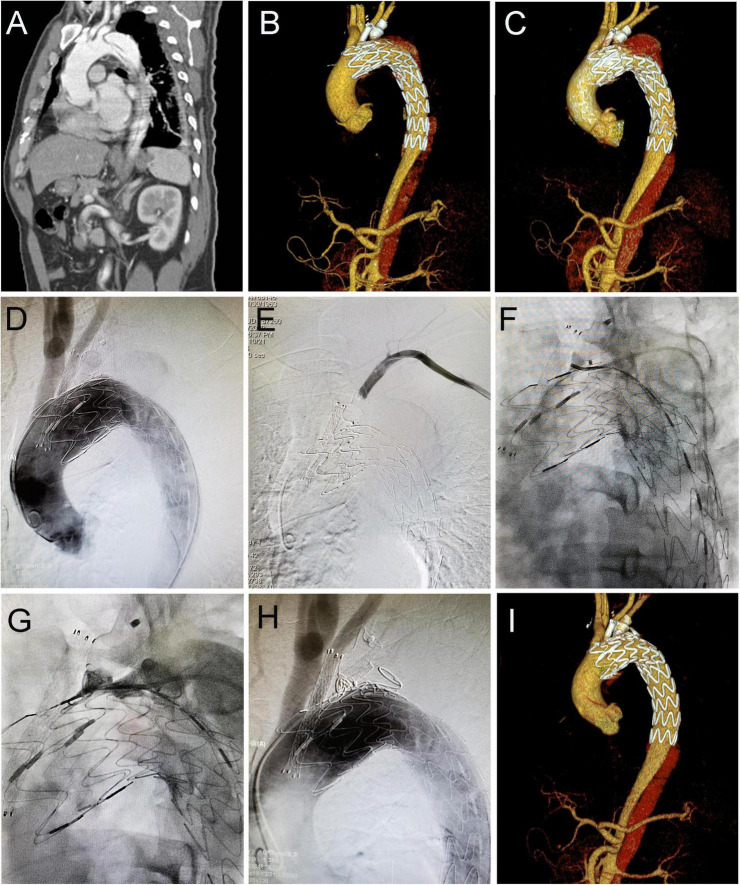
Coil embolization for type IA endoleak 4 years after cTEVAR. Preoperative computed tomography angiography (CTA) showed aortic dissection involving the arch **(A)**. The patient underwent cTEVAR with a chimney stent implanted in LCCA and duct occluder implanted in LSA, and postoperative CTA showed a type IA endoleak **(B)**. The follow-up CTA 4 years later showed the endoleak was significant enlarged **(C)**. With the catheter located in ascending aorta, an angiogram showed contrast could enter the false lumen **(D)**. With the catheter located in LSA, the angiogram showed no contrast entering false lumen **(E)**. With the catheter located in the false lumen, an angiogram showed contrast could enter the aorta **(F)**. After deployment of coils in the false lumen, an angiogram showed contrast could not enter the aorta **(G)**. Post-intervention angiogram **(H)** and CTA **(I)** showed the endoleak completely disappeared. cTEVAR, TEVAR with chimney; CTA, computed tomography angiography; LCCA, left common carotid artery; LSA, left subclavian artery; aRSA, aberrant right subclavian artery.

During follow-up, the type IA endoleak disappeared spontaneously in 14 patients, while three patients with progressively increased type IA endoleak received the secondary intervention (two with coil embolization and one with LSA chimney stent angioplasty). As shown in Figure 2, the patient underwent TEVAR with a chimney stent deployed in LCCA and duct occluder in LSA. He showed progressed type IA endoleak after TEVAR and received coil embolization 4 years later, and then the endoleak totally resolved. The remaining 16 patients with persistent type IA endoleak continued to be monitored with close surveillance. Thus, the rate of persistent and re-intervened type IA endoleak was 5.5% (19/345). Type II endoleak from LSA disappeared spontaneously in two patients, two patients with progressed type II endoleak were re-intervened (one with duct occluder and the other with one more stent deployed in LSA and angioplasty). The rest of the two patients with persistent type II endoleak continued to be managed with conservative treatment.

Besides the five patients having re-intervention for type IA or type II endoleak, three patients underwent secondary TEVAR for distal tear with one more aortic stent deployed (Supplementary Figure 1), three patients received thoracotomy with ascending aorta replacement for type A aortic dissection (Supplementary Figure 2), and one patient underwent IA stent angioplasty 12 months after cTEVAR. The re-intervention rate was 4.1% (14 in 345, including two patients who re-intervened within 30 days after cTEVAR). Retrograde type A aortic dissection occurred in four patients 1, 3, 7, and 18 months after cTEVAR respectively, among whom three underwent open surgery, and one lived uneventfully with conservative treatment. Chimney stent occlusion occurred in five (1.2%) patients, among which two were caused by compression and two by thrombosis. There was one patient found to have a chimney stent in IA compressed and occluded 4 days after TEVAR and he had emergent re-intervention, while another patient was found to have IA chimney stent compression and occlusion 5 years after cTEVAR, and he experienced major cerebral infarction two times but recovered well (Supplementary Figure 3). The other two patients with LSA occlusion (found 1 and 3 years after cTEVAR) lived uneventfully. Partial collapse of IA chimney stents due to compression was observed in two patients 3 and 12 months after cTEVAR. Both of them were asymptomatic, and one was treated conservatively with antiplatelet therapy, while the other was re-intervened with IA angioplasty.

**FIGURE 3:**
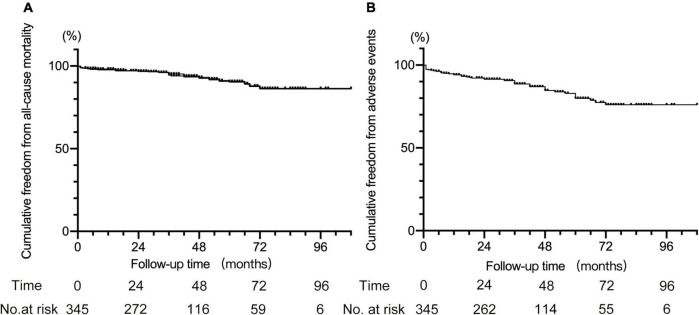
Kaplan–Meier curve for cumulative freedom from all-cause death **(A)** and from adverse events **(B)** after cTEVAR.

For other adverse events, nine patients had major cerebral infarction, among whom three occurred within 30 days after TEVAR. Furthermore, one patient who underwent cTEVAR with IA and LCCA chimney stents deployed had chest pain 42 months after TEVAR. The CTA revealed migration and partial disintegration of the aortic stent (Supplementary Figure 4), and the patient died due to aortic rupture. A total of 23 deaths were recorded, including 4 that occurred within 30 days after TEVAR. The causes of later death after TEVAR included aortic rupture in four, cerebral infarction in two, cerebral hemorrhage in two, myocardial infarction in one, pulmonary infection in one, pulmonary hemorrhage in one, cancer in two, and six patients died for unknown reasons. The all-cause mortality rate was 6.7% (23 in 345), with 5.4% (15 in 278) in patients with single chimneys and 13.3% (8 in 60) in patients with double or triple chimneys (5.4 vs. 13.3%, *P* < 0.05). Collectively, the adverse events occurred in 13.9% of all the patients (48 in 345), and the rate was 11.5% (32/278) in patients with single chimney while 26.7% (16 in 60) in patients with double or triple chimneys (11.5 vs. 26.7%, *P* < 0.05). There was no death or adverse event observed in the seven cases receiving cTEVAR with other techniques. The Kaplan–Meier curves for cumulative freedom from all-cause death and from post-cTEVAR adverse events were shown in Figure 3.

## Discussion

The chimney technique, also known as the parallel stent-graft or double-barrel technique, has been an increasingly popular option in treating aortic arch diseases. A stent-graft deployed parallel to the aortic endograft serves to extend the proximal landing zone and maintain supra-aortic branch patency. Early in 1999, Greenberg et al. implanted a chimney stent to rescue the renal artery during the endovascular repair of an abdominal aortic aneurysm (13). Then in 2002, Criado et al. expanded the application of the chimney technique to preserve an inadvertently covered LSA during TEVAR (8). Compared with other available endovascular techniques such as fenestrated or branched grafts and *in situ* fenestration, the chimney technique offers the advantages of using standard off-the-shelf devices with relatively simple manipulation. Thus, it has been generally accepted in emergent TEVAR and for patients with high surgical risk or challenging arch anatomy. Though there have been abundant publications demonstrating the early and mid-term effects and safety of the chimney techniques, the long-term evidence is still limited (9, 14, 15). To the authors’ knowledge, this study may be the largest case series examining the chimney technique for the preservation of supra-aortic branches during TEVAR with a long period of experience.

The risk of type IA endoleak is the main concern with the chimney technique due to the “gutter” between the chimney stent, the aortic stent-graft, and the aortic wall. The rate of type IA endoleak after cTEVAR is reported to be 10–32% (16–18). However, a study by Ullery et al. showed that 70% of the gutter-related type IA endoleak could resolve spontaneously in 1 year and only 3.3% needed secondary intervention (19). In our series, a total of 38 (11%) patients were found to have immediate-type IA endoleak, among which 5(13%) resolved after repeated balloon molding, 14 (37%) disappeared spontaneously during follow-up, and 3 (8%) received the secondary intervention. A variety of approaches have been proposed to decrease the incidence of type IA endoleak, such as the use of covered chimney stents, enough overlapping between the aortic stent and chimney stent, adequate oversizing of the aortic stent-graft, appropriate angioplasty using the kissing balloon, (11, 20, 21). Recently a novel gutter-free stent-graft for the branch artery, namely Longuette, which consisted of an inner stent and an outer skirt fabric was designed and used for cTEVAR (22). Also, embolizing the gutter with coils or glue is reported to be effective to treat persistent type IA endoleak (23).

In the current study, covered stents were used for chimneys in all patients. However, other groups also reported the use of bare stents (18, 24), and a consensus has not been reached about which is more suitable. In our opinion, when using a bare stent as a chimney, blood flow could enter the gutter and proximal landing zone *via* the mesh of the stent, while covered stents can seal the flow and form a blind end to the gutter, thus decreasing the risk of endoleak around the chimney stent. However, some of the covered stents had to be implanted by surgical access because of their large delivery system; whereas, with a thin delivery system, the bare stent can be implanted by percutaneous radial artery access (17). Further studies are needed to compare the bare stent and covered stent used in cTEVAR and provide guidance for reasonable choice.

The patency of the chimney stent is another important issue for the chimney technique. Previous studies have shown that the primary patency of chimney stents could be as high as 98–99% (14, 25). The current study also showed a high patency rate of chimney stents, and only six patients experienced chimney stent occlusion or stenosis. One had acute compression and occlusion of IA chimney stent and received re-intervention emergently. One had chronic compression and occlusion of IA chimney stent and experienced two major strokes. While two patients with LSA thrombosis lived uneventfully during follow-up. In addition, two other patients were observed to have IA chimney stent stenosis due to compression. Though occlusion or stenosis of chimney stent in LSA is mostly asymptomatic and does not require re-intervention (17), it is critical for the chimney stent in IA or LCCA to remain patent and provide sufficient cerebral blood perfusion.

A cerebrovascular accident is a significant risk of aortic arch repair, as there were vital supra-aortic branches supplying to the brain. Moulakakis et al. reported the rate of stroke for hybrid surgery was 7.6% (26), and Tazaki et al. reported periprocedural stroke rate for TEVAR with branched stent-graft could be 16% (7). As cTEVAR has less manipulation in the aortic arch and targets supra-aortic branches, it has been shown to have a lower incidence of cerebrovascular events compared with other techniques (18, 27). In the current study, nine (2.6%) patients had cerebral infarction during follow-up, among which three occurred within 30-day after cTEVAR, and two patients died of cerebral infarction. Retrograde type A aortic dissection (RAAD) is another important complication of the chimney technique. The rate of RAAD after TEVAR was estimated to be 1.6–2.5% by previous studies (28, 29), and its occurrence was related to extent of oversizing, proximal bare stent, and angulation of the arch. The interactive force of the chimney stent and the aortic stent-graft on the aortic wall in cTEVAR potentially increased the incidence of RAAD. In the current study, a total of four patients (1.2%) developed RAAD 1, 3, 7, and 18 months after cTEVAR respectively, among whom three underwent open surgery.

The double and triple chimney techniques in the aortic arch are still controversial and were reported to pose a high risk. Previous studies have shown that double and triple chimney techniques were found to be with a higher instant endoleak rate compared with single chimneys (18, 20), which is related to the wider gutter formed in double and triple chimney techniques. Recently, Guo et al. reported a series of 31 patients receiving cTEVAR with double and triple chimney techniques, in which the rate of intraoperative type IA endoleak was more than 30% and the overall mortality rate was 16.1% (30). In this study, the patients with double and triple chimney techniques were found to have a higher incidence of type IA endoleak (8.6 vs. 20% *P* < 0.05), a higher rate of adverse events after the operation (11.5 vs. 26.7%, *P* < 0.05) and higher all-cause mortality (5.4 vs. 13.3%, *P* < 0.05) compared with single chimney technique. Moreover, two cases with early re-intervention due to migration or compression of chimney stents were both cases treated with the double chimney technique, but these events only occurred in our early stage of practice with cTEVAR. Based on our experience, the reasonable allocation of chimney stents is crucial for double and triple chimney techniques. Sometimes, implanting snorkel stents in LSA should be considered to avoid crowding of chimney stents in the proximal landing zone.

There are several limitations to this study. First, it was a retrospective and observational study with heterogeneous groups of patients, and the outcomes only represented experiences obtained at two institutions. Second, as the study only included those receiving cTEVAR, we did not make a comparison of the outcomes between the chimney technique, open surgery, hybrid procedure, and fenestrated or branched stent-grafts. Third, as some patients did not have adequate imaging follow-up, it was not possible to assess chimney patency and evolution of endoleak accurately.

## Conclusion

Thoracic endovascular aortic repair with the chimney technique provides a minimally invasive alternative with good chimney graft patency and low postoperative mortality during follow-up. However, re-interventions are not unusual after cTEVAR, and there are still concerns about the post-cTEVAR complications such as immediate-type IA endoleak, neurological morbidities, chimney stent compression, and occlusion, and retrograde type A aortic dissection. The double and triple chimney techniques should be used judiciously as they have a higher risk for type IA endoleak and adverse events after operation compared with the single technique.

## Data availability statement

The raw data supporting the conclusions of this article will be made available by the authors, without undue reservation.

## Ethics statement

The studies involving human participants were reviewed and approved by Ethics Committee of the Second Xiangya Hospital and Fuwai Hospital. The patients/participants provided their written informed consent to participate in this study.

## Author contributions

JL, CS, AD, QL, MLi, HH, and XL contributed to conception and design of the study. YX, SL, LS, LW, TW, KF, and MLu collected the data and organized the database. YX and SL performed the statistical analysis and interpretation. JL, YX, and SL wrote the first draft of the manuscript. All authors contributed to manuscript revision and approved the submitted version.
