# Supplementary material for: Outcomes of thoracic endovascular aortic repair with chimney technique for aortic arch diseases
Source: Front Cardiovasc Med. 2022 Aug 4;9:868457. doi: 10.3389/fcvm.2022.868457 (PMC9386043; doi:10.3389/fcvm.2022.868457)
Supplement: Supplementary file 1 [file Data_Sheet_1.docx]

**SUPPLEMENTARY FIGURES**


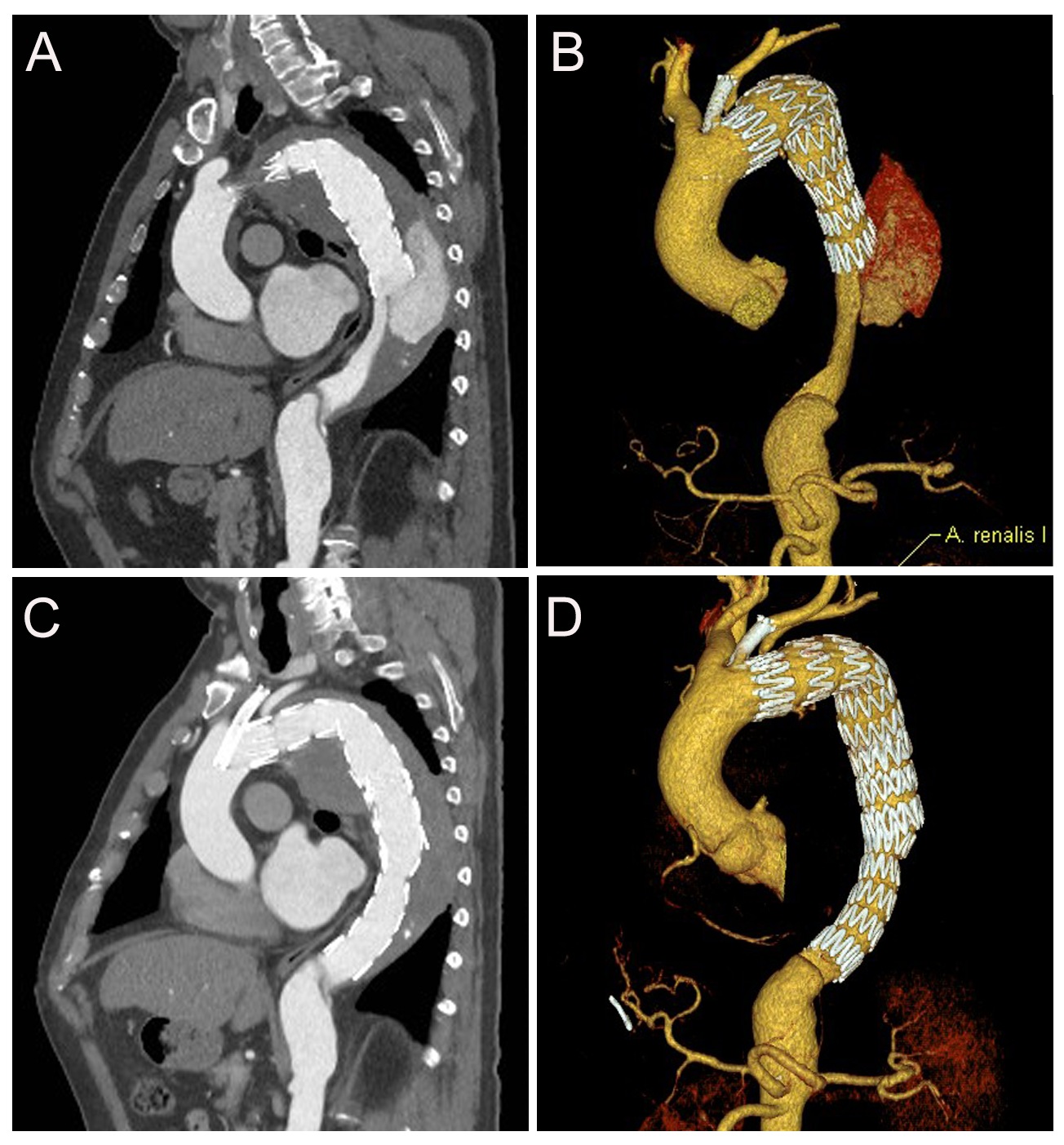


Figure S1 Reintervention for (stent graft-induced new entry tear, SINE) 5 years after cTEVAR with a chimney stent in LCCA. The sagittal view and reconstruction picture of CTA showed the distal tear and significant enlargement of false lumen (A, B). The patient received secondary intervention with one more aortic stent graft implanted and the distal tear was covered (C, D).


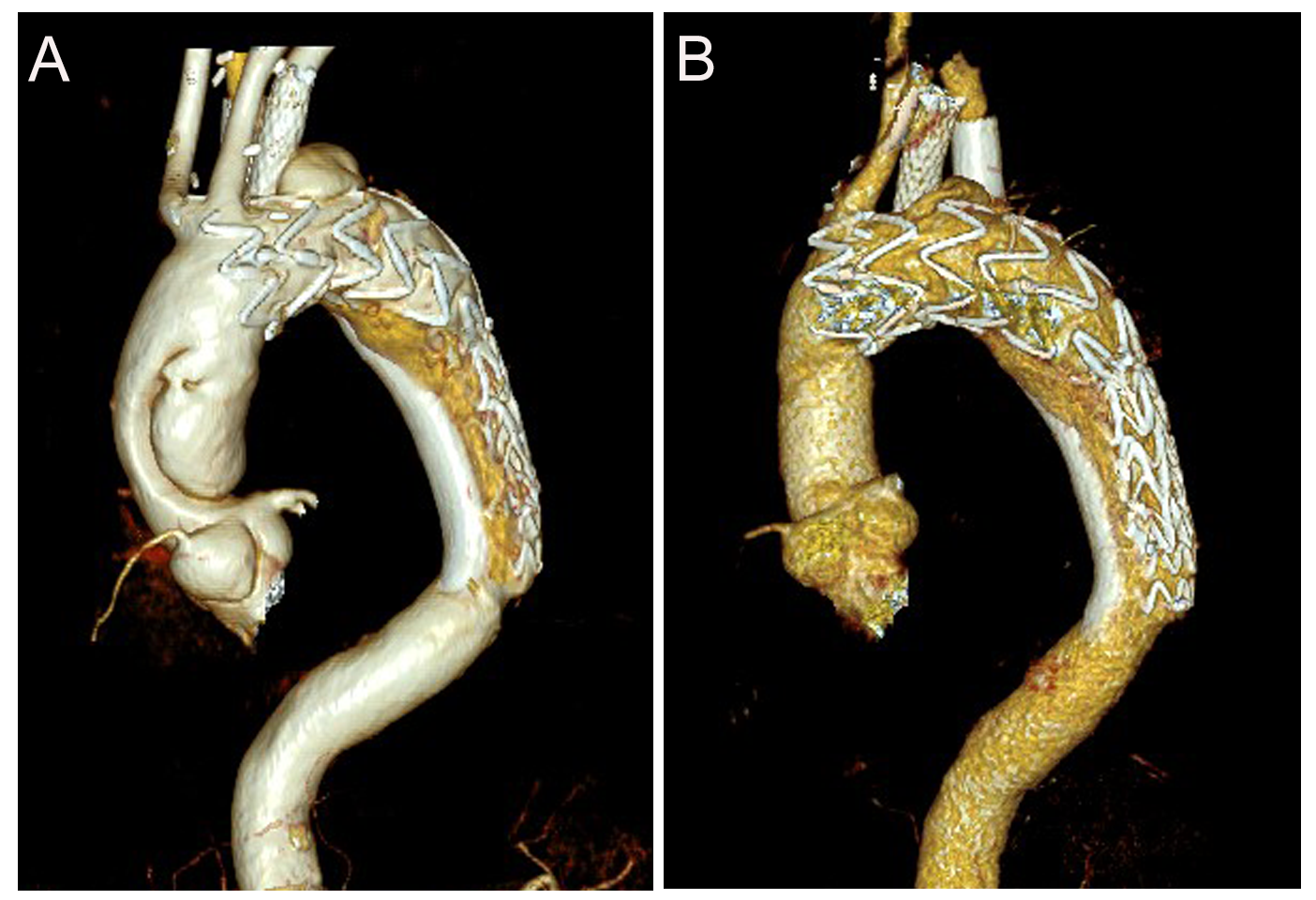


Figure S2 Retrograde type A aortic dissection 17 months after cTEVAR with chimney stent in LSA and snorkel stent in aRSA. CTA showed the retrograde type A aortic dissection (A). The patient underwent open surgery with replacement of ascending aorta (B).


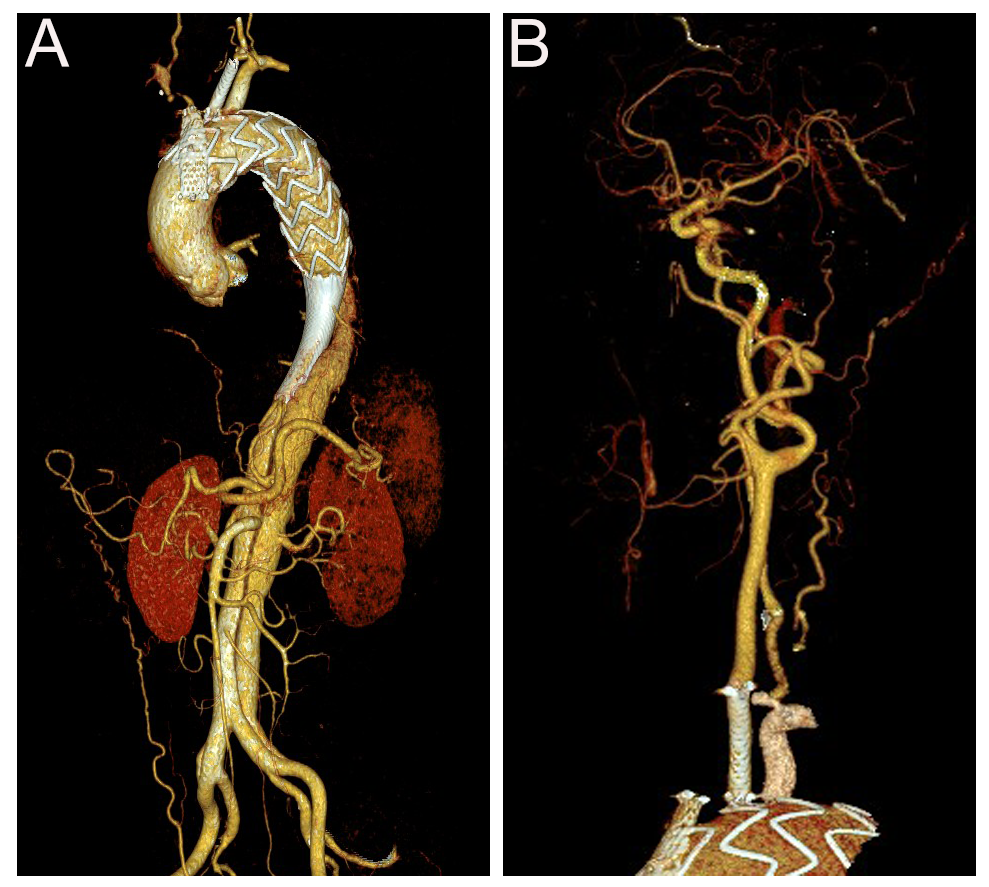


Figure S3. IA stent compression and occlusion 5 years after cTEVAR with chimney stents in IA and LCCA. CTA showed IA stent compression and occlusion 5 years after cTEVAR (A, B). The patient experienced 2 episodes of major stokes but recovered well, and he was treated conservatively with antiplatelet therapy.


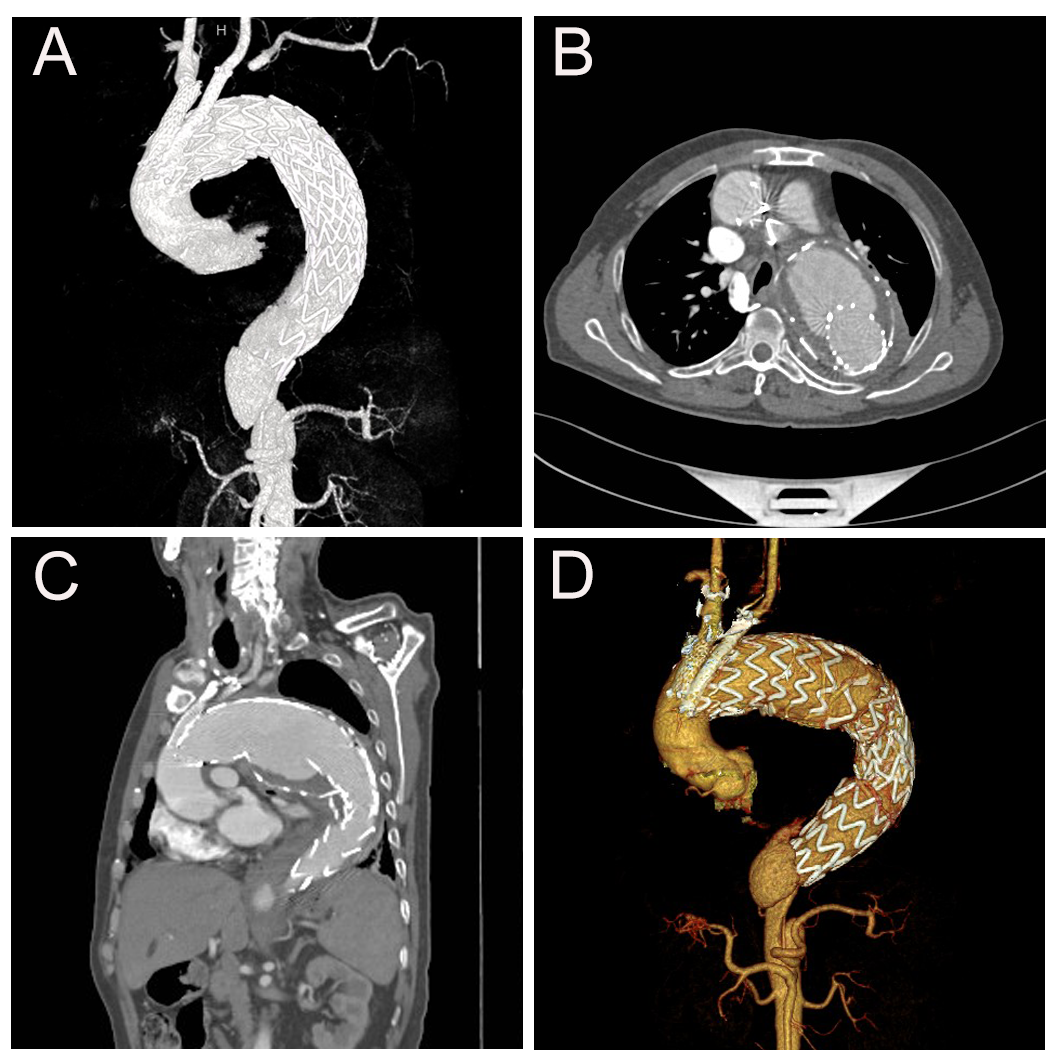


Figure S4. Migration and disintegration of aortic stent graft 42 months after cTEVAR with chimney stents in IA and LCCA. The patient with the diagnosis of aortic dissection received cTEVAR with chimney stents in IA and LCCA (A). He had abruptly chest pain 42 months later, and CTA revealed migration and disintegration of aortic stent, and the patient died possibly due to aortic rupture (B, C, D).
